# Hierarchical H-ZSM5 zeolites based on natural kaolinite as a high-performance catalyst for methanol to aromatic hydrocarbons conversion

**DOI:** 10.1038/s41598-019-54089-y

**Published:** 2019-11-26

**Authors:** Ahmad Asghari, Mohammadreza Khanmohammadi Khorrami, Sayed Habib Kazemi

**Affiliations:** 1 Department of Chemistry, Faculty of Science, Imam Khomeini International University, Qazvin, 3414896818 Iran; 2 Department of Chemistry, Institute for Advanced Studies in Basic Sciences(IASBS), Zanjan, 45137-66731 Iran; 3 Center for Research in Climate Change and Global Warming (CRCC), Institute for Advanced Studies in Basic Sciences (IASBS), Zanjan, 45137-66731 Iran

**Keywords:** Heterogeneous catalysis, Porous materials

## Abstract

The present work introduces a good prospect for the development of hierarchical catalysts with excellent catalytic performance in the methanol to aromatic hydrocarbons conversion (MTA) process. Hierarchical H-ZSM5 zeolites, with a tailored pore size and different Si/Al ratios, were synthesized directly using natural kaolin clay as a low-cost silica and aluminium resource. Further explored for the direct synthesis of hierarchical HZSM-5 structures was the steam assisted conversion (SAC) with a cost-effective and green affordable saccharide source of high fructose corn syrup (HFCS), as a secondary mesopore agent. The fabricated zeolites exhibiting good crystallinity, 2D and 3D nanostructures, high specific surface area, tailored pore size, and tunable acidity. Finally, the catalyst performance in the conversion of methanol to aromatic hydrocarbons was tested in a fixed bed reactor. The synthesized H-ZSM5 catalysts exhibited superior methanol conversion (over 100 h up to 90%) and selectivity (over 85%) in the methanol conversion to aromatic hydrocarbon products.

## Introduction

Zeolites are materials with versatile applications in ion-exchange membranes^1^, chemical separation^2^, gas sorption^3^, and catalysis^4^. They are composed of aluminosilicate structures in which tetrahedral T atoms (T = Si, Al, etc.) are connected to oxygen atoms, constituting a porous framework structure^5,6^. Among the zeolite materials, ZSM-5 has attracted particular attention owing to its tunable acidity, high specific surface area, as well as excellent ion-exchange ability and shape selectivity^7,8^. Owing to such recognizable features, ZSM-5 is an efficient catalyst in the methanol to hydrocarbon conversion process (MTH). MTH process on zeolite catalysts is a well-known industrial process first discovered by Mobil company in 1979. MTH products are essential raw materials for downstream petrochemical industries such as oil and polymers^8,9^. A current problem in MTH process with conventional ZSM-5 catalyst is the fast deactivation of ZSM-5 due to the rapid coke deposition on Bronsted acidic sites and diffusion limitation in the microporous structure of ZSM-5^10^. In this regard, designing zeolites with pores larger than micropores and reducing crystal size in order to shorten the diffusion length could be good strategies to developing more efficient catalysts. In particular, creating a secondary pore system consisting of mesopores (2–50 nm) inside the microporous zeolite crystals is a good approach to fabricating materials with efficient transport pathways for molecules^11^. In recent years, many researchers have focused on hierarchical zeolites that, owing to their joint micro and mesopore structure, possess high stability and selectivity^12^. So far, hierarchization has been mostly based on dieselization^13^ and dealumination^14^, introducing hard^15^ and soft^16^ templating methods or some combination thereof^17,18^. Nevertheless, dealumination and desilication are destructive synthesis methods which can damage the ZSM-5 zeolite structure^19^. Moreover, large scale production of hierarchical ZSM-5 zeolites for MTH application is still a challenging task because high quantities of organic mesopore-directing templating agents need to be used, entailing high manufacturing costs. More importantly, considering HSE (health, safety, and environment) upon template removal, it is necessary to vacate the porosity of the final material^20^. Clays are a good natural resource of aluminium and silica in the economical synthesis of ZSM-5 for catalytic application^21^. Although there are many reports on the use of clays as Al/Si precursor, there has been little progress in directly producing the hieratical H form of ZSM-5 zeolites from costless natural resources such as kaolinite. In addition, various mesopore agents have been used for the construction of hierarchical ZSM-5 structures, among which monosaccharides and disaccharides such as glucose and sucrose are low-cost mesoporogens which can be adapted into a green approach to introducing secondary mesopores in the zeolite structure^22,23^. Furthermore, nano/microsized ZSM-5 has been fabricated by several hydrothermal methods such as organic solvent system^24,25^, or without a solvent^15,26^ using aluminosilicate gel as the raw precursor material in the Na^+^–Tetra Propyl ammonium hydroxide system. In this system, Na^+^ ions are maintained in the framework of zeolites for charge compensation^20^. For most applications in which ZSM-5 is used as a solid acid catalyst, the H-form of the zeolite is favorable, hence the necessity for NaZSM-5 to be exchanged with the ammonium salt in order to obtain HZSM-5 catalysts. However, the ion exchange process suffers from several problems such as high cost, reduced production efficiency, and significant energy consumption. Accordingly, the direct synthesis of H-ZSM-5 using ammonium hydroxide to replace alkali metal cations and eliminate the ion exchange process can be advantageous over the conventional methods. Bibby *et al*. successfully synthesized HZSM-5 in a NH_4_^+^-TPA system without using any alkali metal cations^27^. In another work, Xuchen Lu *et al*. directly synthesized highly crystalline HZSM-5 zeolite materials by a steam-assisted conversion (SAC) method without alkali metal ions. They used kaolin as the precursor material, and the catalyst was used in methanol to olefin conversion (MTO)^28^. More recently, Lin-Bing Sun *et al*. reported on the direct synthesis of a ZSM-5 zeolite through SAC method using a natural clay, attapulgite (ATP)^29^.

In this work, the steam assisted conversion (SAC) was used as a facile and scalable synthesis method for the direct synthesis of hierarchical H-ZSM5 from natural kaolin clay as a low-cost silica and aluminium resource. Furthermore, for the first time, high fructose corn syrup was used as a green, abundant, and inexpensive material to introduce secondary pores (mesopore agent) to the system. High fructose corn syrup is a clear aqueous solution of saccharides and can be considered as a good and inexpensive source of sugar. Finally tested was the performance of H-ZSM5 as a catalyst in methanol to hydrocarbon process. The catalytic results illustrated exceptional stability and selectivity to aromatic compounds.

## Results and Discussion

**Figure 1:**
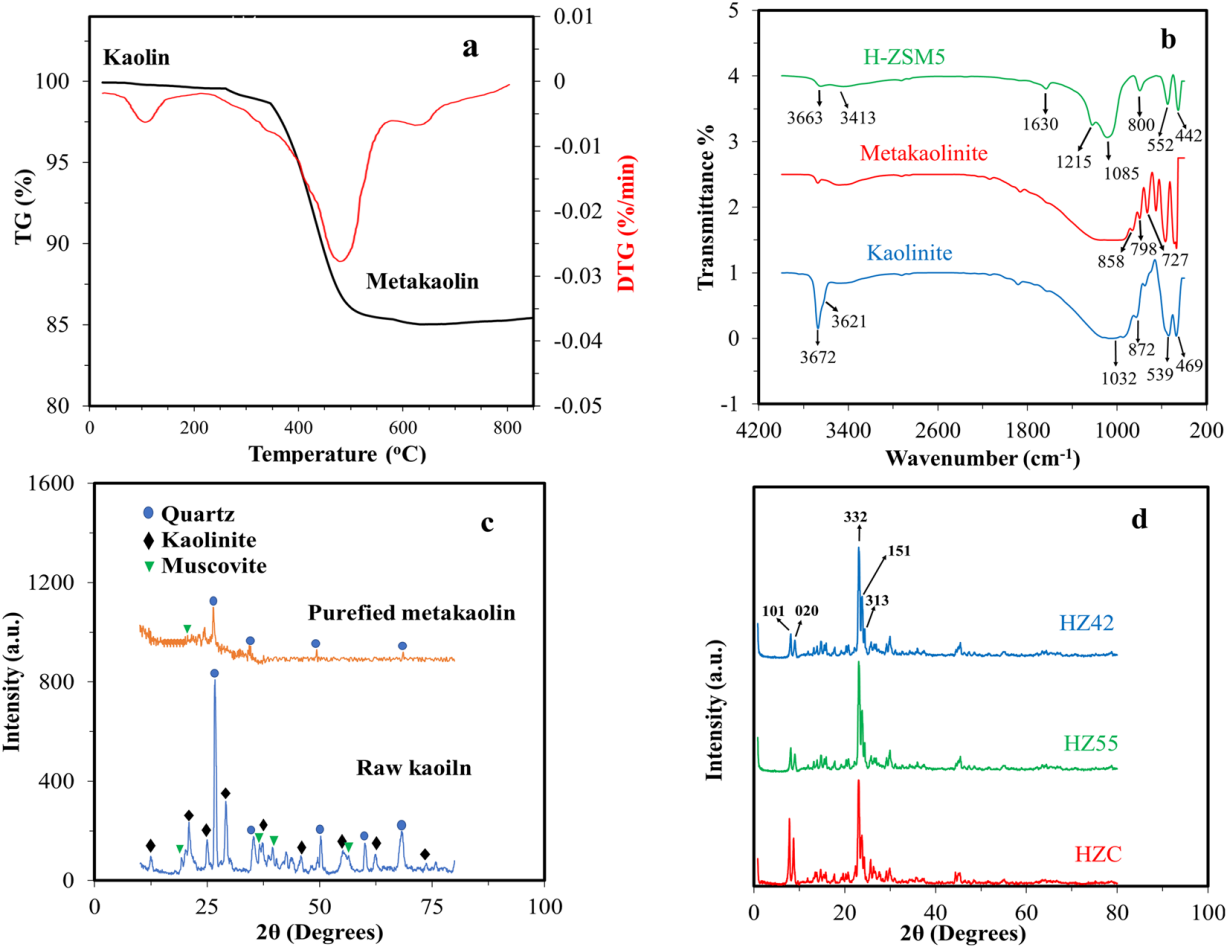
(**a**) TG/DTG plot of purified kaolinite. (**b**) FT-IR spectra of kaolin, metakaolin and zeolite H-ZSM5. (**c**) XRD patterns of raw kaolinite and purified meta-kaolinite. (**d**) XRD comparison of synthesized zeolites.

TGA/DTG profile of kaolin sample are shown in Fig. 1a. The total weight loss according to the TG curve was 14.8%. The slight weight loss observed in the temperature range of 40–180 °C can be attributed to the loss of adsorbed water. The significant mass loss in the temperature range of 400–700 °C is associated to dehydroxylation of the structural Al-OH groups resulting from kaolin to metakaolin conversion. Moreover, no obvious mass loss can be observed in the temperature range of 750–850 °C. The process of the thermal metakaolinization of kaolin is represented by the following reaction^30,31^;

1 $${{\rm{Al}}}_{{\rm{2}}}{{\rm{Si}}}_{{\rm{2}}}{{\rm{O}}}_{{\rm{5}}}{(\mathrm{OH})}_{{\rm{4}}}\mathop{\longrightarrow }\limits^{{\rm{400}}^\circ C-{\rm{750}}^\circ C}{{\rm{Al}}}_{{\rm{2}}}{{\rm{O}}}_{{\rm{3}}}{{\rm{.SiO}}}_{{\rm{2}}}{+\mathrm{2H}}_{{\rm{2}}}{\rm{O}}({\rm{g}})$$

**Figure 2:**
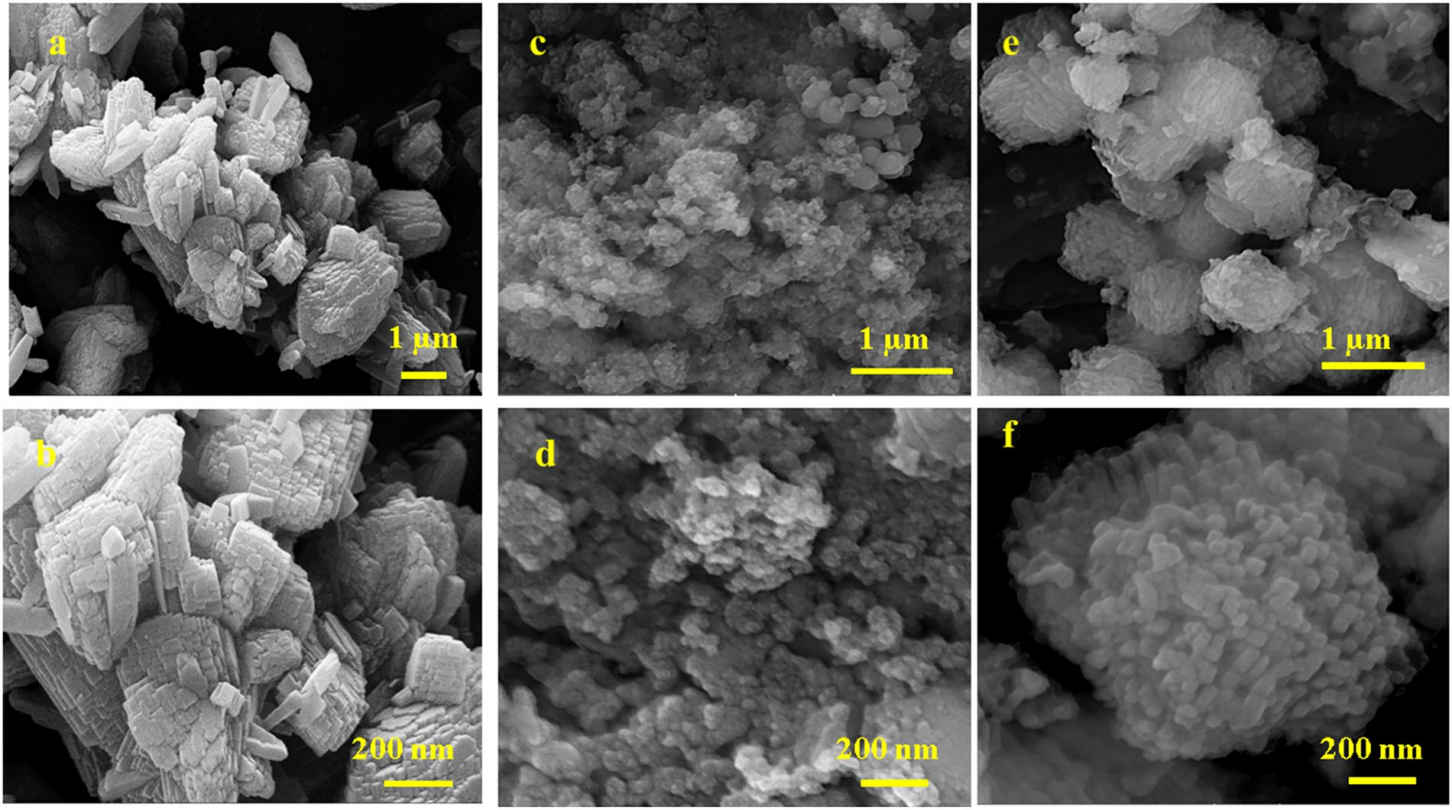
FE-SEM images of synthesized zeolites at two different magnifications. (**a–b**) HZC. (**c–d**) HZ55. (**e–f**) HZ42.

**Figure 3:**
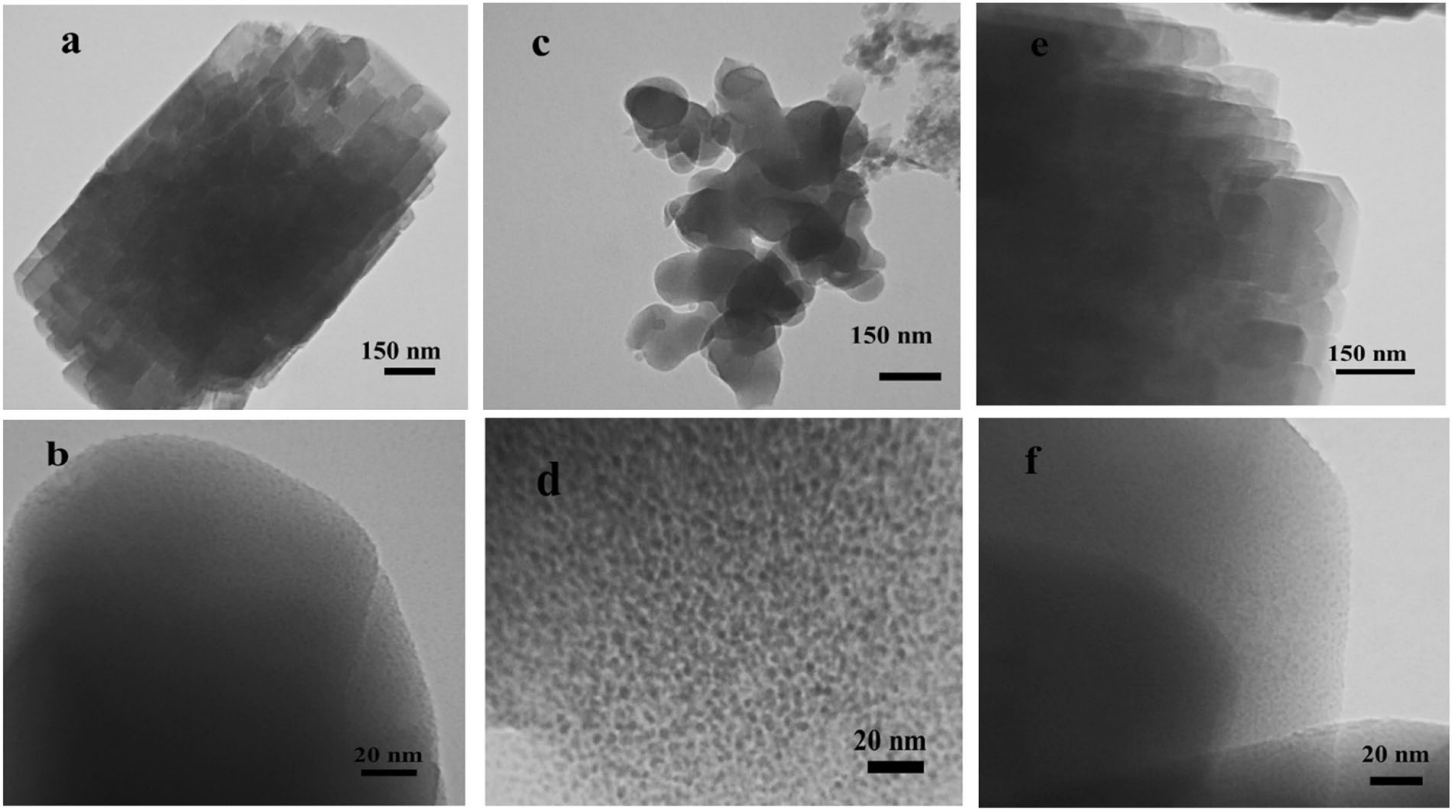
TEM images of synthesized catalysts. (**a–b**) HZC. (**c–d**) HZ55. (**e–f**) HZ42.

**Figure 4:**
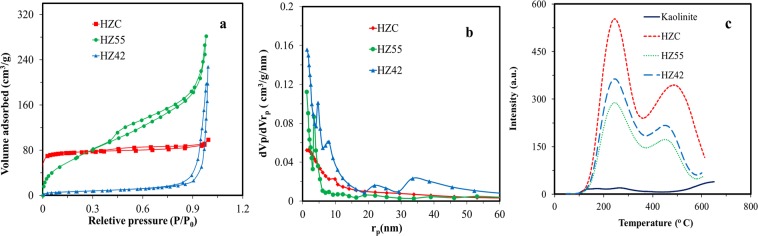
(**a**) N_2_ adsorption/desorption isotherms of hierarchical zeolite HZC, HZ55 and HZ42. (**b**) BJH plot for HZC, HZ55, and HZ42. (**c**) NH_3_-TPD analysis of catalyst samples.

FT- IR spectroscopy is a powerful method for identifying characteristic structural bonds in zeolite materials such as ZSM-5^32^. Figure 1b shows the FT-IR spectra of kaolin, metakaolin, and H-ZM5 Zeolites. The characteristic bands for kaolin, including hydroxyl bands (3492–3672 cm^−1^), Al-O-Al (870–913 cm^−1^), SiO_2_ (471, 1032 and 1062 cm^−1^), and Si-O-H (539 cm^−1^), were observed. More precisely, the bands at 3672 and 3621 cm^−1^ are related to stretching vibrations of the inner-surface hydroxyl (Al-OH) groups and the stretching vibration of the inner hydroxyl groups^33,34^. For metakaolin, the band at 798 cm^−1^ is due to the conversion of Al^3+^ octahedral kaolin to tetrahedral metakaolin coordination^35^. In addition, there are no sharp bands in the hydroxyl region (3492–3672 cm^−1^) indicating the formation of metakaolin. The spectrum of H-ZSM5 shows bands at (1085−1100 cm^−1^) and (798−810 cm^−1^) from the zeolite framework, i.e., the asymmetric and symmetric vibration modes of T−O−T (T = Si and Al) groups. The broad but distinct vibration band at (542−552 cm^−1^) is attributed to the asymmetric stretching mode of the double-five ring in ZSM-5. The band at 444−452 cm^−1^ is ascribed to the tetrahedral Si−O bending mode^29^. Also, the broad band at 3413 cm^−1^ and the sharp peak at 1635 cm^−1^ are attributed to the structural hydroxyl groups and bending mode of physically adsorbed water, respectively. Figure 1c compares the XRD patterns of raw kaolinite and purified meta stable metakaolinite phase. The raw kaolinite shows a typical diffraction pattern with a characteristic d_001_ value of 0.71 nm. As we can see, the kaolin peaks not be seen in XRD pattern of metakaolin, this phenomenon shows the conversion of the crystalline structure of kaolinite phase to amorph metakaolinite phase. Also, the peaks at 2Ɵ of 27, 34.47, 49.35 and 68.95° are related to trace amount of quartz impurity in metakaolin sample. For further investigation, the percentage of chemical composition in impure raw kaolinite and purified kaolinite is given in Table S1. Also, XRD pattern of synthesizes zeolites are shown in Fig. 1d. Two characteristic peaks at 2Ɵ of 7–10 and 23–25° (JCPDS Card No. 01–079–2401) confirm the formation of H-ZSM5^36^. Scherrer equation was used to calculate the crystal size from the high-intensity peaks at 2Ɵ of 7.93° with characteristic d_101_ value of 1.12 nm. The average crystal sizes of 47.4, 36.1, and 37.4 nm were estimated for HZC, HZ55, and HZ42, respectively. Figure 2 shows the structure and morphology of the optimized H-ZSM5 zeolites with different magnifications recorded by FE-SEM. HZC appears as aggregated crystals (Fig. 2a,b) and HZ55 (Fig. 2c,d) shows a nano-spherical particle approximately 11 to 40 nm connected each other to make larger spherical morphology (Fig. S1a). The HZ42 shows rod shapes supporting each other to make oriented nanorod aggregated structures (Fig. 2e,f). The diameter of each rod is 26 to 45 nm for HZ42 (see supplementary Fig. S1b online). The TEM images of the synthesized samples are shown in Fig. 3 (a-b for HZC, c-d for HZ55 and e-f for HZ55). As we can see, all three samples show clearly a porous structure. The N_2_ physisorption measurements in Fig. 4a show a specific surface area of 283.5, 389.6, and 345 m^2^/g for HZC, HZ55, and HZ42, respectively. The increase at P/Po < 0.05 and a hysteresis at P/Po = 0.4–0.9 seen in the three synthesized samples reveal the existence of both micro and mesopores in the samples; however, IV type isotherm curve with a significant uptake at P/Po = 0.8 to 1 demonstrates the presence of developed mesopores in the sample HZ55. The t-plot method was used to distinguish micro- and meso-porosity. With the introduction of HFCS (HFCS55 and HFCS42, respectively) agent and the increase in the Si/Al ratio, the N_2_ physisorption specific surface areas, total pore volume (V_total_) and mesopore volume increased dramatically concerning HZ55 and HZ42 (see Table 1). The Barrett-Joyner-Halenda (BJH) method was used to assess pore size distribution, which showed an average size of 4.5, 6.9, and 9.8 nm for HZC, HZ55, and HZ42 samples, respectively (Fig. 4b). NH_3_-TPD test was carried out to determine the density and strength of acid sites (as shown in Fig. 4c). Two obvious desorption peaks around 200–300 C and 400–500 C are attributed to weak and strong acid sites, respectively^23,37^. With the increase in Si/Al ratio and decrease in crystal size, a reduction occurred in general acidity comprised of weak and strong acid sites. However, the high acidity of microporous H-ZSM5 zeolite (HZC) is due to the large amount of acid sites incorporated in micropores^28^. More quantitative information regarding weak and strong acid sites is given in supplementary Table S2 online. As a result, introducing HFCS as a directing agent in gel composition, improve the mesopore volume with a larger pore size as regards HZ55 and HZ42 in comparison with HZC. Moreover, moderate total acidity was obtained through increasing Si/Al ratio, reducing crystal size (increasing amorphous nature) and augmenting mesoporous area. To obtain the catalytic activities, the methanol conversion was calculated for the tests based on the following equation:

2 $$Methanol\,Conversion\,( \% )=\frac{{M}_{in}-{M}_{out}}{{M}_{in}}\times 100$$

where *M*_*in*_ and *M*_*out*_ are the amounts of pumped methanol in the reactor and unconverted methanol in aqueous samples, respectively.

**Table 1:** Textural properties of synthesized H-ZSM5 samples.

Sample	Si/Al^a^ ratios	S_BET_^b^ (cm^3^/gr)	S_micro_ (cm^3^/gr)	S_ext_^c^ (cm^3^/g)	V_total_ (cm^3^/gr)	V_micro_ (cm^3^/gr)	V_meso_^d^ (cm^3^/gr)	d_p_ (nm)	Average crystal size (nm)
HZC	36.8	283	191	92	0.16	0.12	0.04	4.5	47.4
HZ55	41.6	389	203	186	0.6	0.37	0.23	6.9	36.1
HZ42	48.1	345	216	129	0.51	0.3	0.21	9.8	37.4

^a^Determined by XRF.

^b^Calculated with the BET model.

^c^Determined by the t-plot method, S_ext_ = S_BET_ − S_micro_.

^d^Determined by the t-plot method, V_meso_ = V_total_ − V_micro_.

The selectivity to the main product was calculated from Eq. 3:

3 $$Selectivity\,( \% )=\frac{{H}_{i}}{{H}_{t}}\times 100$$

*H*_*i*_ is the amount of the product of interest and *H*_*t*_ is the total amount of hydrocarbon products calculated by GC/MS instrumental analysis. For further efficiency evaluation, the yielded percentage as a total liquid organic phase in the product can be quantitatively calculated using the following equation:

4 $$Yield\,( \% )=\frac{{M}_{org}}{{M}_{org}+{M}_{aq}}\times 100$$

where M_org_ and M_aq_ are the weight of liquid organic and aqueous phase respectively.

**Figure 5:**
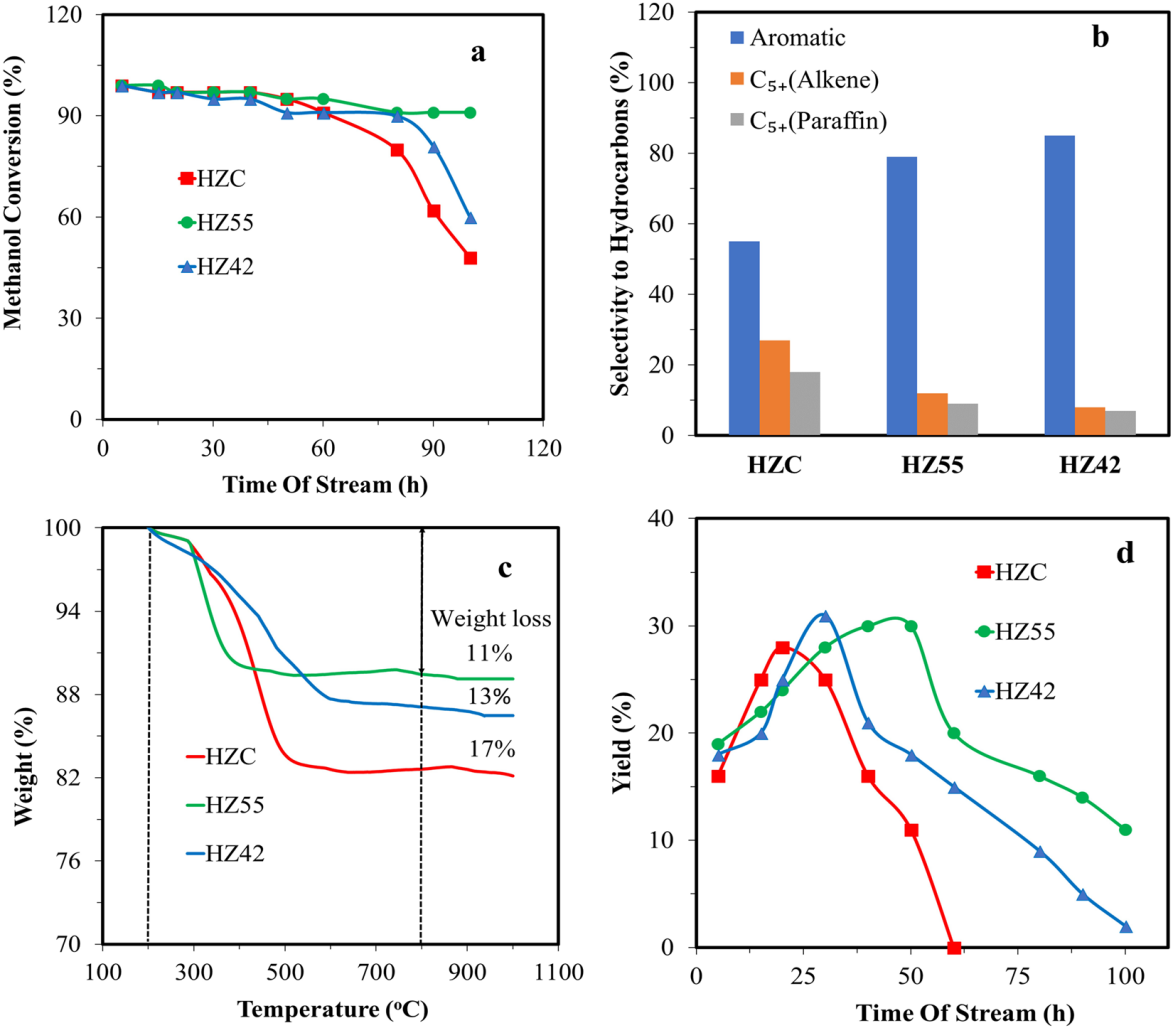
(**a**) Methanol Conversion for HZC, HZ55, and HZ42. (**b**) Selectivity to hydrocarbons for each catalyst. (**c**) TGA analysis of coke deposited on catalysts following MTH reaction. (**d**) The yield of liquid organic products for HZC, HZ55, and HZ42.

Figure 5a illustrates the methanol conversion against time of stream for the three different synthesized zeolites. In the first 15 h period, the conversion efficiency is about 100% for all catalysts. If we consider that catalyst deactivation occurs when the methanol conversion efficiency is below 90%, HZC deactivates during the first 50 hours before deactivation of the other two catalysts. This phenomenon could be due to the large number of micropores, large crystal size compared to the mesopores in the structure of HZC, so that the micropores encompass most of the acidic sites. Furthermore, HZ55 and HZ42 remained active for methanol conversion over considerably longer periods. HZ55 showed an activity period of 100 hours, which could be due to a large number of mesopores, moderate total acidity and lowest crystal size compared to conventional zeolite (HZC). Another interesting point is that, in HZ55 the fraction of meso and micropores is balanced, which is very desirable for high-performance zeolite catalysts. On the other hand, catalyst deactivation is related to coke or products adsorption, smaller crystal size improve diffusion, thus decrease deactivation by coke. Another possibility is the concentration (and distribution) of acid sites that should contribute to tertiary reaction leads to more coke formation. Figure 5b shows the product distribution of hierarchical Zeolite samples. HZC, in comparison with HZ55 and HZ42, showed more selectivity to C_5+_ alkene (27%) and paraffin (18%). For HZ55 and HZ42, product distribution shifted from C_5+_ alkene and alkane toward larger aromatic and poly aromatic hydrocarbons with the increase in the pore size from 4.2 nm to 6.9, 9.8 nm and also with the increase in the Si/Al ratio. This is because relatively large molecules are involved in aromatization and cyclization reactions; hence requiring larger pore for their diffusion^38^. Moreover, the relative increase in larger isomerized aromatic and poly aromatic hydrocarbons for HZ55 and HZ42 (Table 2) arises from major active sites confined inside the mesopores, hence the good change of side reactions before cracking large molecules or blocking the cavities.

**Table 2:** Percentage of some aromatic in organic products determined by GC/Mass^a^.

Sample	Benzene	Xylene	Other larger isomerized aromatic and poly aromatics
HZC	0.3	14	40.7
HZ55	0.5	18.8	59.7
HZ42	0.3	16	68.7

^a^Reaction conditions: Time on stream 10 h, 390 °C, 1 atm, WHSV = 10 h^−1^.

Figure 5c shows that the amounts of coke formed in the samples decreased significantly in HZ55 and HZ42 among the samples, indicating that stronger acidic sites of the catalyst make the coke formation happen more quickly. The higher stability of HZ55 and HZ42 catalysts can be ascribed to their moderate acid density and high mesoporous volume with a tailored pore size that results in a smoother catalytic reaction; therefore, there is lower diffusion resistance due to lower coke formation, and the coke formed inside the micropores can easily exit the pores due to the short diffusion path lengths. The net result is less coke accumulation inside the micropores, which is in accordance with TGA thermograms. In microporous catalysts such as HZC, the migration of coke precursors is not facile, and deposition of coke will occur inside the micropores, destroying the catalyst by blocking the micropores^39,40^. The coke formation process also increased by the higher acid density in the HZC catalyst. These two parameters are mostly responsible for lowering the catalyst life time. Figure 5d indicates the yield of liquid organic products in the methanol conversion process for the three synthesized zeolites. The maximum yields for HZC, HZ55, and HZ42 were 28%, 31%, and 30% respectively. By comparison, after 40 h, the yield of the mentioned zeolites was reduced down to 16, 30, and 21% from their highest yield point, corroborating the better catalytic efficiency of HZ55 and HZ42. Table 3 shows the superior performance of the synthesized catalyst (HZ55) and compares their structures in this wok with previously reported works in literature. In comparison with the reported ZSM-5 catalysts^22,41–44^, the hierarchical H-ZSM5 catalyst (HZ55) in the present research showed improved catalytic activity over an extended period due to moderate acidity, high specific surface area, tailored pore size which is comparable with catalysts reported in the previous literature. In addition, the synthesized catalysts showed high selectivity of around 79% (for HZ55) and 85% (for HZ42) regarding aromatic compounds. The catalytic results revealed that the hierarchical H-ZM5 can be used as an efficient catalyst for methanol to aromatic hydrocarbon process.

**Table 3:** Comparison of present work with other reported ZSM-5 catalysts.

Sample	S_BET_	V_meso_	Total acidity	Weak acidity	Strong acidity	Deactivation time (Me conversion under 90%)	Selectivity to aromatics	Reference
ZSM-5	272.2	0.06	1.19	0.48	0.71	42	72.5	22
ZSM-5	332.7	0.1	1.11	0.54	0.57	98	75.1	22
ZSM-5	365	0.56	0.4	0.06	0.22	—	30	39
ZSM-5	378	0.09	0.779	0.392	0.387	40	86	40
ZSM-5	321	0.12	1.34	0.5	0.84	70	66.9	41
Zn/ZSM-5	331	0.2	0.56	0.209	0.182	98	48	42
**ZSM-5**	389	0.23	0.727	0.427	0.3	100	79	**Present work**
**ZSM-5**	345	0.21	0.856	0.48	0.376	60	85	**Present work**

## Conclusions

In summary, a facile SAC route was used in the direct synthesis of hierarchical H-ZSM5 zeolites from natural clay. High fructose corn syrup was used as a green and efficient secondary meso pore agent in a gel composition. The zeolites were used as efficient catalysts in the MTH process. The hierarchical H-ZSM5 catalyst showed improved catalytic activity with the decrease in the coking rate over an extended period, compared to conventional zeolite. Furthermore, the synthesized zeolites exhibited enhanced selectivity of around 85% for aromatic compounds. Our approach to the facile and scalable fabrication of hierarchical H-ZM5 zeolites has a great potential for use in the logical design of many industrial catalytic applications.

## Methods

The chemical reagents used in the study include: methanol (HPLC grade), tetra propylammonium hydroxide (40% in water), tetraethyl orthosilicate (TEOS, reagent grade, 98%), and ammonium hydroxide (NH_4_OH, 25%) from Merck Company (Germany) without any further treatment; high fructose corn syrup (HFCS55 with Fructose = 55%, Glucose = 42%, maltose, and other oligosaccharide = 3% and HFCS42 with Fructose = 42%, Glucose = 52%, maltose, and other oligosaccharide = 6%) was prepared from Glucosan Company (Iran), and Iranian natural kaolinite clay was used as Al and Si source.

The nanocrystalline Hierarchical ZSM-5 zeolites were synthesized using Iranian kaolin clay as Si and Al precursors. The Iranian natural kaolin was first heated at 800 °C for 3 h to obtain metakaolin which was de-aluminated by leaching in sulfuric acid solution with a ratio of 1:5 solid to acid. After the mixture was stirred for 5,6, and 7 h, it was filtered, washed by distilled water, and finally dried. The dealuminated metakaolin with a ratio of Si/Al 36.8, 41.6, and 48.1 was used in the preparation of the gel composite of 1SiO_2_: 0.039–0.0135 Al_2_O_3_: 0.08 TPAOH: 5H_2_O with 2 g HFCS55 (HZ55) and HFCS42(HZ42). Next, the gel was transferred into 50 ml autoclave (in which ammonia was underneath) for steam-assisted crystallization at 180 °C under autogenous pressure for 24 h. The obtained zeolites were finally calcinated at 550 °C for 6 h to remove the template. For comparison, a conventional microporous zeolite with a Si/Al ratio of 36.8 was used, named HZC. The HZC zeolite synthesized by same procedure but without HFCS. Figure S2 shows the typical autoclave for the steam assisted synthesis procedure.

The kaolin and H-ZSM5 samples were characterized by X-ray fluorescence (XRF) conducted on a Bruker S4 Explorer instrument in order to determine their chemical compositions. FTIR measurements were performed on a Bruker Vertex 80 in the range of 400 to 4000 cm^−1^ with a resolution of 4 cm^−1^. Samples were made into transparent pellets by mixing in appropriate amounts of KBr.The morphology of the hierarchical H-ZSM5 zeolites was investigated by a Field-Emission Scanning Electron Microscope (MIRA3TESCAN-XMU, Zeiss Sigma). The crystal structure of the synthesized H-ZSM5 was studied by their XRD patterns recorded with a PW1730 Philips instrument (the Netherlands) with Cu Ka radiation (40 kV, 30 mA). Nitrogen gas adsorption measurements were carried out at −196 °C on a BELSORP-MINI II gas/vapor adsorption instrument (BEL Co, JAPAN). The samples were vacuumed at 300 °C for 6 h before conducting the adsorption measurements. NH_3_-TPD analysis was recorded by a Micromeritics Chemisorb 2720 with a thermal conductivity detector. About 200 mg of the sample was used for all samples. NH_3_ adsorption measurements were recorded at 25 °C under an atmosphere of 5% NH_3_–He, and their desorption measurements were recorded from a temperature of 150 °C to 650 °C at a heating rate of 10 °C min^−1^ in He atmosphere.

The liquid organic samples were analysed by 7890B Gas Chromatography (GC, Agilent Co, USA) connected to 5977B Mass Spectroscopy (MS) and equipped with a split/splitless. GC-MS analysis was carried out on a HP-5MS capillary column with a dimension of 30 m × 0.25 mm and a film thickness of 0.25 µm. The mass spectrometer was operated in the electron impact ionization mode with an ionizing energy of 70 eV. The interface and ion source temperatures were set at 300 °C. The aqueous phases of the products were analysed by GC Younglin (model 6500) with a FID detector and bonded polystyrene-divinylbenzene (DVB) PLOT-Q Megabore column (30 m × 0.53 mm) with a film thickness of 40 µm. The total amount of coke residue following the reaction was measured by Thermogravimetric analyser (TG, Model Q600, TA Co, USA) from a temperature of 30 to 1000 °C under air flow with a heating rate of 10 °C min^−1^.

The catalytic performance of the catalysts was evaluated in a fixed bed reactor with a 12 mm inner diameter. Typically, 1 g of the prepared catalyst (mesh 20–30) was used in the centre of the reactor. The N_2_ flow (88 mL min^−1^, inlet pressure 5 bar) passed into the reactor at 300 °C for 2 h; catalytic reaction was then carried out with methanol feeding 0.2 ml.min^−1^ at 390 °C for 100 h after which, the reactor outlet was withdrawn. It consisted of organic, aqueous and gaseous samples which were separated. The organic phase was then analysed by GC/MS chromatography, and the aqueous phase was analysed by a gas chromatograph equipped with a FID detector and PLOT-Q capillary column, respectively.

## Supplementary information

Supplementary Information

## Data Availability

The datasets generated during and/or analyzed in the current study are available from the corresponding author on reasonable request.
