# Supplementary material for: Hierarchical H-ZSM5 zeolites based on natural kaolinite as a high-performance catalyst for methanol to aromatic hydrocarbons conversion
Source: Sci Rep. 2019 Nov 26;9:17526. doi: 10.1038/s41598-019-54089-y (PMC6879614; doi:10.1038/s41598-019-54089-y)
Supplement: Supplementary file 1 — Supplementary Information [file 41598_2019_54089_MOESM1_ESM.docx]

**Hierarchical H-ZSM5 zeolites based on natural kaolinite as a high-performance catalyst for methanol to aromatic hydrocarbons conversion**

Ahmad Asghari *^a^, Mohammadreza Khanmohammadi Khorrami ^a^ and Sayed Habib Kazemi ^b,c^

*^a^ Chemistry Department, Faculty of Science, Imam Khomeini International University, Qazvin 3414896818, Iran*

*^b^ Department of Chemistry, Institute for Advanced Studies in Basic Sciences(IASBS), Zanjan 45137-66731, Iran*

*^c^ Center for Research in Climate Change and Global Warming (CRCC), Institute for Advanced Studies in Basic Sciences (IASBS), Zanjan 45137-66731, Iran*

**Table S1.** Percentage of chemical composition in

impure raw kaolin and purified kaolin ^a^.

|  | SiO_2_ | Al_2_O_3_ | TiO_2_ | Na_2_O | K_2_O | Fe_2_O_3_ |
| --- | --- | --- | --- | --- | --- | --- |
| Kaolin | 73 | 21 | 0.98 | 0.38 | 0.3 | 1 |
| Purified Kaolin | 56 | 45 | 0.06 | 0.09 | 0.01 | 0.08 |

^a^ measured by XRF.

**Table S2**. NH_3_-TPD data of different synthesized catalysts.

| **Samples** | **Temp (°C)** |  | **Acidity (mmolNH_3_/g)^a^** |  | **Total** |
| --- | --- | --- | --- | --- | --- |
|  |  | **Weak** | **Temp (°C)** | **Strong** |  |
| HZ1  HZ2 | 200-300  200-300 | 0.866  0.427 | 400-500  400-500 | 0.581  0.3 | 1.447  0.727 |
| HZ3 | 200-300 | 0.480 | 400-500 | 0.376 | 0.856 |

^a^ The concentration of strong acid sites and weak acid sites Analyzed by NH_3_-TPD.


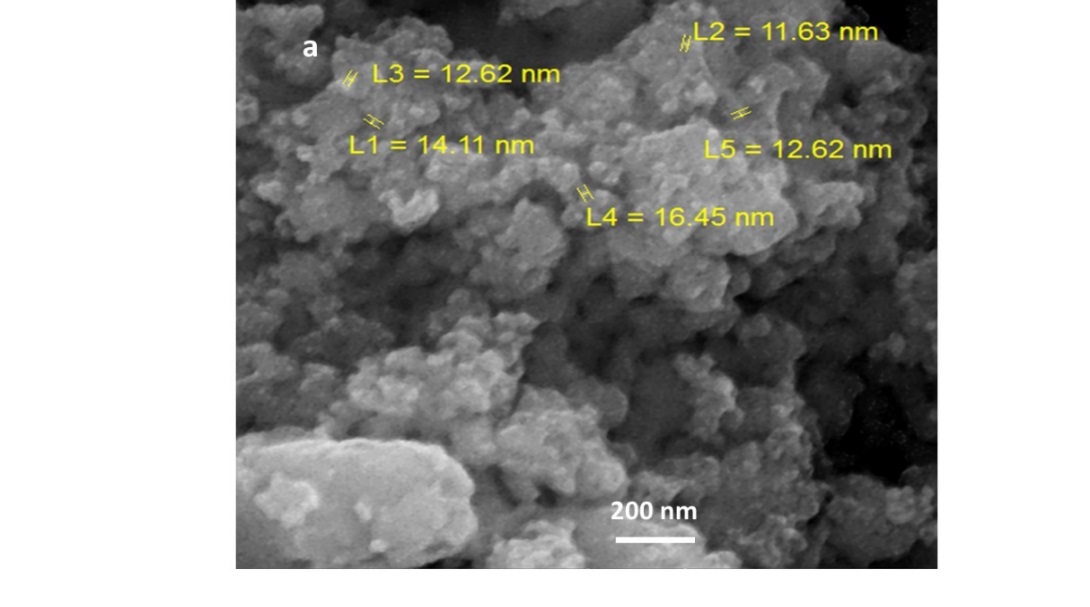

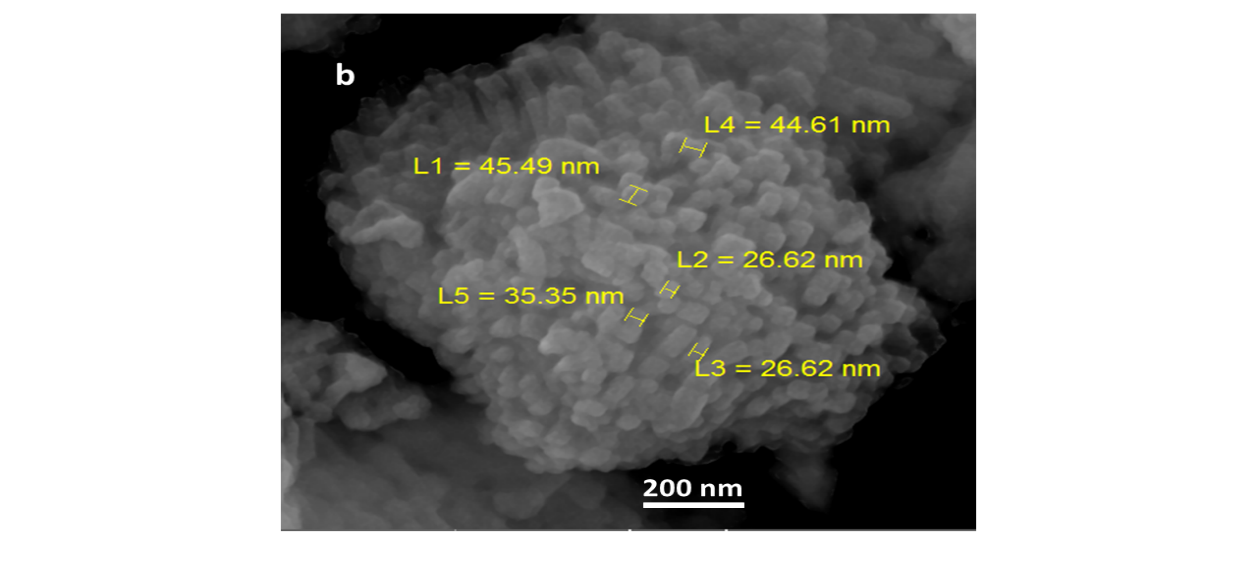


**Figure S1.** SEM images for synthesized catalysts (a) HZ55 (b) HZ42.


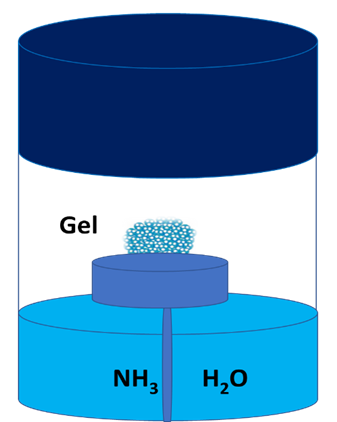


**Figure S2**. Steam assisted synthesis procedure.
